# Effect of the Combination of *Clostridium butyricum* and Mycelium of *Phellinus igniarius* on Intestinal Microbiota and Serum Metabolites in DSS-Induced Colitis

**DOI:** 10.3390/nu16010153

**Published:** 2024-01-02

**Authors:** Rou Zhao, Qiaoyi Zhang, Ting Huang, Yun Tian, Guiping Guan, Yuanshan Lin

**Affiliations:** 1 College of Bioscience and Biotechnology, Hunan Agricultural University, Changsha 410128, China; rosezhao@stu.hunau.edu.cn; 2 Agricultural Bioengineering Institute, Changsha 410128, China; annezhang@stu.hunau.edu.cn (Q.Z.); evehuang@stu.hunau.edu.cn (T.H.); tianyun@hunau.edu.cn (Y.T.); guanguiping@hunau.edu.cn (G.G.)

**Keywords:** *Clostridium butyricum*, mycelium of *Phellinus igniarius*, colitis, intestinal flora, serum metabolome

## Abstract

*Clostridium butyricum* (CB) and *Phellinus igniarius* (PI) have anti-inflammatory, immune regulation, anti-tumor, and other functions. This study aimed to explore the therapeutic effect of CB and mycelium of PI (MPI) alone and in combination on colitis mice induced by dextran sodium sulfate (DSS). Mice were randomly assigned to five groups: (1) control (CTRL), (2) DSS, (3) CB, (4) MPI, and (5) CB + MPI (CON). The weight of the mice was recorded daily during the experiment, and the length of the colon was measured on the last day of the experiment. The colons were collected for hematoxylin and eosin staining, colon contents were collected for intestinal flora analysis, and serum was collected for metabolite analysis. The results showed that compared with the DSS group, CB, MPI, and CON treatments inhibited the weight loss and colon length shortening caused by DSS, significantly increased the concentrations of interleukin (IL)-4, IL-10, and superoxide dismutase, and significantly decreased the concentrations of IL-6, tumor necrosis factor-α, and myeloperoxidase. Gene sequence analysis of 16S rRNA showed that CB, MPI, and CON treatments changed the composition and structure of intestinal microorganisms. Metabolome results showed that CB, MPI, and CON treatments changed serum metabolites in DSS-treated mice, including dodecenoylcarnitine, L-urobilinogen, and citric acid. In conclusion, CB, MPI, and CON treatments alleviated DSS-induced colitis in mice by regulating intestinal flora and metabolites, with the CON group having the best effect.

## 1. Introduction

Ulcerative colitis (UC) is a complex chronic inflammatory disease that belongs to inflammatory bowel disease (IBD), mainly located in the rectum and colon [1]. Currently, the specific pathogenesis of UC is still unclear—genetic, environmental, microbial factors, and immune disorders are believed to be related to the pathogenesis of UC [2,3,4]. In general, UC results from abnormal immune activation owing to interactions between the host and gut microbiota [4]. The dysregulation of intestinal homeostasis not only affects the metabolic pathway and immune system, promoting intestinal inflammation, but also increases the permeability of the intestine, allowing intestinal bacterial translocation, which aggravates the inflammatory response [2,3]. At present, the drugs used to treat UC are amino salicylic acid, steroids, antibiotics, and immunosuppressants [5]. However, the therapeutic effectiveness of these drugs is limited, and patients may develop resistance. Thus, there is a need to determine a new regimen to treat UC.

Supplementation with probiotics is a crucial approach to regulate disturbances of intestinal flora. Some probiotic strains have been shown to regulate the disruption of the gut microbiota and reduce intestinal damage and inflammation [6,7]. *Clostridium butyricum* (CB) is an important probiotic that enhances the intestinal barrier and regulates immunity and inflammation. The role of CB in intestinal injury, IBD, colorectal cancer, and other diseases has been widely reported [8,9]. CB produces short-chain fatty acids (SCFAs) during fermentation [10]. SCFAs have anti-inflammatory effects and can inhibit the activation of nuclear factor-κB (NF-κB) macrophages by inhibiting the activity of inflammatory mediators in the intestinal epithelium [11]. CB can also maintain the expression of tight junction protein and has a protective effect on the epithelial barrier [12].

*Phellinus igniarius* (PI), which is a basidiomycete in the family *Hymenochaetaceae*, is a functional fungus known as “Sanghuang” in China [13]. Research has predominantly focused on the active components in the fruiting body of PI, and there are few studies on the mycelium of PI (MPI) [14]. The large-scale fermentation of metabolites or enzyme preparations will result in a large number of cell residues, but how to use mycelium reasonably and reduce environmental pollution has become a problem. Mycelium has good flavor, high-quality protein, and essential micronutrients that bring benefits to public health [15]. The mycelium of *Cordyceps sinensis*, obtained by fermentation, has been widely used in developing various drugs, mainly for treating chronic kidney disease [16]. Lu et al. found through animal experiments that the mycelia of *Antrodia camphorata* and *Armillariella tabescens* have protective effects on the liver [17]. There are many active components in the MPI, which have many functions, such as anti-oxidation, anti-tumor, and immune regulation, but the cost of extracting these components is high [18,19]. In this study, MPI was used directly to treat colitis, and CB and MPI were used in combination. MPI not only acts as a nutrient but also provides suitable ecological conditions for anaerobic CB to enter the intestine, helping CB exert its health properties.

The purpose of this study is to investigate the repair function of CB and MPI alone or in combination on the dextran sodium sulfate (DSS)-induced mouse colitis model and examine the effects on the intestinal flora and serum metabolites in order to provide a reference for the development of colitis-related diseases.

## 2. Materials and Methods

### 2.1. Materials

The *Clostridium butyricum* BJ-10 and *Phellinus igniarius* SH-1 were obtained from the Institute of Agricultural Bioengineering of Hunan Agricultural University. CB was cultured at 37 °C for 15 h. A certain volume of the bacterial culture was centrifuged, the supernatant was removed, and then the bacteria were washed with sterile water and placed in a freeze-dryer to produce freeze-dried CB powder.

After being cultured in 100-L and fermented for 8 days, the fermentation liquid of PI was filtered with 300 mesh non-woven filter. The filtrate was used for polysaccharide preparation, the MPI was harvested and washed three times with sterile water, then broken into pieces and placed in a freeze-dryer. The powdered MPI was obtained from freeze-dried MPI through a 100-mesh sieve. Before each intragastric administration of mice, CB powder and MPI powder were dissolved in sterile physiological water.

### 2.2. Experimental Design

**Figure 1:**
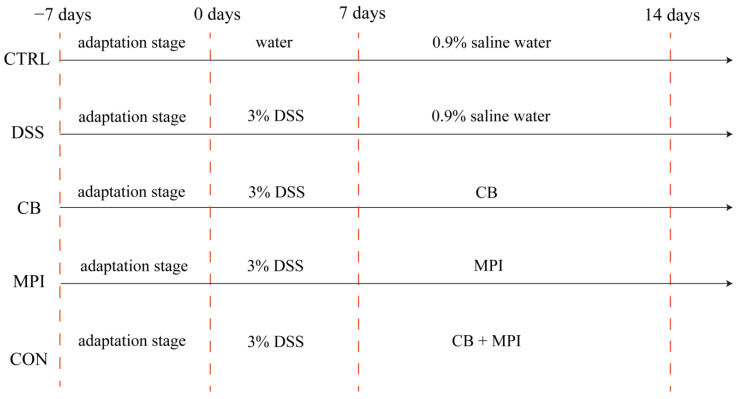
Design and grouping of mice colitis model.

Animal experiments were performed on the basis of the Guidelines for Care and Use of Laboratory Animals of Hunan Agricultural University. Fifty ICP female mice (Slaughter Jingda Co., Changsha, China), 8 weeks old and weighing an average of 20 g, were placed in a room with a temperature of 24 ± 1 °C and a 12-h cycle of darkness and light. During the experiment period, the mice could drink water and feed at will. After 7 days of adaptation, the mice were randomly assigned to five groups (*n* = 10 per group): CTRL, DSS, CB, MPI, and CON (Figure 1). From days 0 to 7, mice in the CTRL group were given drinking water, whereas mice in the other groups were given drinking water supplemented with 3% (*w*/*v*) DSS (molecular weight 36,000–50,000 Da; Dalian Meilun Biotechnology Co., Ltd., Dalian, China) to induce UC. From days 8 to 14, mice in the CTRL group and DSS group were given 0.9% normal saline by intragastric gavage; the CB group was gavaged with CB 1 × 10^6^ colony forming units (CFU); the MPI group was gavaged with MPI 300 mg/kg; and the CON group was gavaged with CB 5 × 10^5^ CFU and MPI 150 mg/kg.

On day 15, all mice were prevented from feeding for 12 h. Subsequently, blood was taken from the eyeball after anesthesia with ether, then the mice were dissected, the colon and the colon contents were placed at −80 °C, and serum was collected and refrigerated after centrifugation for serum metabolite analysis.

### 2.3. Histological and Biochemical Analysis

Following fixation, the colon was embedded in paraffin, sectioned, and stained with hematoxylin and eosin (H&E). The sections were then dehydrated with anhydrous alcohol, sealed with a neutral sealant, and observed under a microscope (Nikon, Tokyo, Japan). Histological activity index (HAI) scoring of the colon sections was performed using the evaluation criteria listed in Table S1.

The concentrations of interleukin (IL)-4, IL-6, IL-10, tumor necrosis factor-α (TNF-α), superoxide dismutase (SOD), and myeloperoxidase (MPO) in colon contents were detected by enzyme-linked immunosorbent assay (ELISA) kits (Table S2).

### 2.4. Microbial Community Analysis

Analysis of the microbes in the colon contents of the five groups of mice was performed according to the methods of Tan et al., with some modifications [20]. DNA was extracted from the samples using a TGuide S96 Magnetic Soil/Fecal DNA Kit (Tiangen Biotechnology (Beijing) Co., Ltd., Beijing, China). Primers 338F (5′-ACTCCTACGGGAGGCAGCA-3′) and 806R (5′-GGACTACHVGGGTWTCTAAT-3′) were used to amplify the 16S rRNA hypervariable region V3–V4. The PCR products were detected by agarose gel electrophoresis, purified products were collected, and the paired ends was performed on the Illumina NovaSeq 6000 platform.

### 2.5. Serum Metabolome Analysis

A serum sample of 100 μL was taken, and the extraction solution (methanol:acetonitrile = 1:1) of 500 μL was added and vortex mixed for 30 s. Samples were incubated at −20 °C, centrifuged at 12,000× *g* rpm for 15 min, supernatant was taken, and then placed in a vacuum concentrator. The dried metabolites were redissolved, mixed, and underwent ultrasound for 10 min. The sample was centrifuged again, the supernatant was placed in an injection bottle and then tested. The liquid chromatography–mass spectrometry (LC/MS) system for metabolomics analysis is composed of Waters Acquity I-Class PLUS ultra-high performance liquid tandem Waters Xevo G2-XS QTof high-resolution mass spectrometer. The electrospray ionization (ESI) ion source parameters were as described by Zhang et al. [21].

### 2.6. Data Analysis

The results are presented as means ± standard deviation (SD). One-way analysis of variance and Duncan’s post hoc test were used to test statistical significance (SPSS 22 software, IBM, Chicago, IL, USA). SPSS 22 was used for bivariate Pearson correlation analysis of the data. Origin 2021 (Origin Lab, Northampton, MA, USA) was used to draw graphs such as those for inflammatory cytokines and to produce correlative heat maps. Images were edited with Adobe Illustrator 2021 (Adobe, San Jose, CA, USA). * *p* < 0.05 indicates a significant difference.

## 3. Results

### 3.1. H&E Staining of Mice Colons

**Figure 2:**
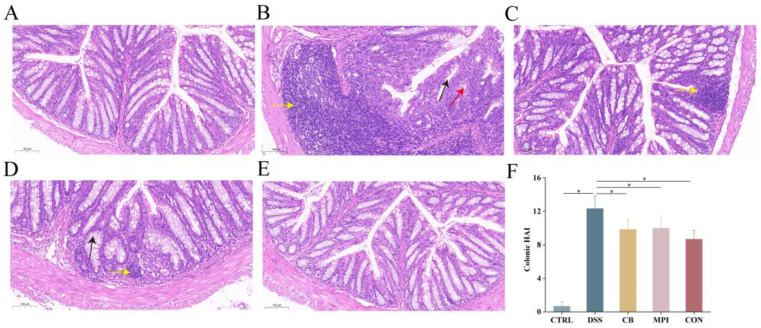
Therapeutic effect of CB, MPI, and CON on colon tissue injury. The red arrow represents goblet cells. The black arrow represents crypts. The yellow arrow represents inflammatory cells. (**A**) CTRL, (**B**) DSS, (**C**) CB, (**D**) MPI, (**E**) CON, and (**F**) HAI score of colonic tissues. * *p* < 0.05.

In this experiment, all mice in the CTRL, CB, MPI, and CON groups survived, but two mice in the DSS group died, and the final survival rate was 80%. The colonic staining results of the different groups of mice are shown in Figure 2A–E. The colonic structure in the CTRL group was intact without damage. The colons in the DSS group showed significant pathological features of colitis, including severe deformation or absence of crypts (black arrow), a reduced number of goblet cells (red arrow), and infiltration of inflammatory cells (yellow arrow). Compared with the DSS group, the degree of colon injury decreased after CB, MPI, and CON treatment, especially the degree of recovery of crypts. Besides, as shown in Figure 2F, the HAI scores of the CB, MPI, and CON groups were significantly lower than the DSS group (*p* < 0.05). These results indicated that CB, MPI, and CON could repair DSS-induced colonic tissue injury. The CON group exhibited the optimal effect among them.

### 3.2. Effects of CB, MPI, and CON on Body Weight and Colon Length in Colitis Mice

**Figure 3:**
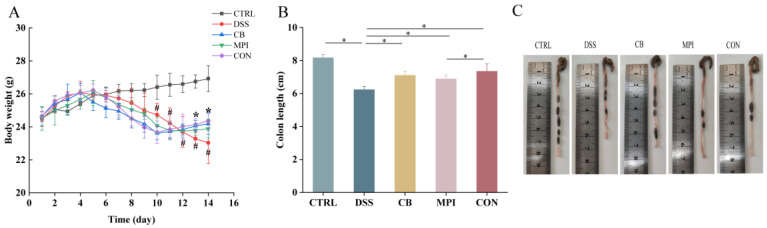
The effects of CB, MPI, and CON on body weight and colon length in mice. (**A**) Weight change in each group; (**B**) changes of colon length in each group; (**C**) colon chart in each group of mice. Compared with CTRL, # *p* < 0.05; compared with DSS, * *p* < 0.05.

Weight loss and shortened colon length are characteristics of colitis. Therapeutic effects of CB, MPI, and CON on body weight and colon length in inflammatory mice were monitored and measured, respectively. The weight of the colitis mice is shown in Figure 3A. After 4 to 5 days of adding 3% DSS to the drinking water, mice began to lose weight, while the weight of the mice increased after 5 days of treatment by CB, MPI, and CON (*p* < 0.05). The colon length in DSS group was shorter than that in CTRL group as shown in the figure (Figure 3B,C and Figure S1), while the colon length in CB, MPI, and CON groups were significantly longer than that in DSS group in final measurement (*p* < 0.05). These results showed that CB, MPI, and CON treatments could recover weight loss and colon length reduction in colitis mice. In particular, the effects of the CON group were better than those of the CB and MPI groups.

### 3.3. Effects of CB, MPI, and CON on the Activities of Inflammatory Cytokines and Enzymes in Colitis Mice

**Figure 4:**
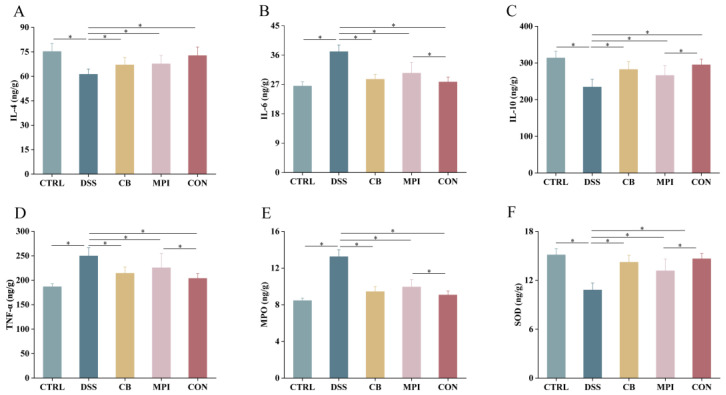
Concentrations of inflammatory cytokines and enzymes in the colon of each group of mice; (**A**) IL-4, (**B**) IL-6, (**C**) IL-10, (**D**) TNF-α, (**E**) MPO, and (**F**) SOD. * *p* < 0.05.

Inflammatory cytokines are crucial in the course of IBD, and excessive inflammatory responses can damage the colon (Figure 4). Compared with the CTRL group, the concentrations of IL-4, IL-10, and SOD were significantly decreased, and the concentrations of IL-6, TNF-α, and MPO were significantly increased in the DSS group (*p* < 0.05). However, compared with the DSS group, in the CB, MPI, and CON groups, the concentrations of IL-4, IL-10, and SOD were significantly increased, and the concentrations of IL-6, TNF-α, and MPO were significantly decreased (*p* < 0.05). The results showed that CB, MPI, and CON had the effect of restoring the imbalance of inflammatory cytokines. The effect of the CON group was the most significant.

### 3.4. Effects of CB, MPI, and CON on Intestinal Microorganisms

**Figure 5:**
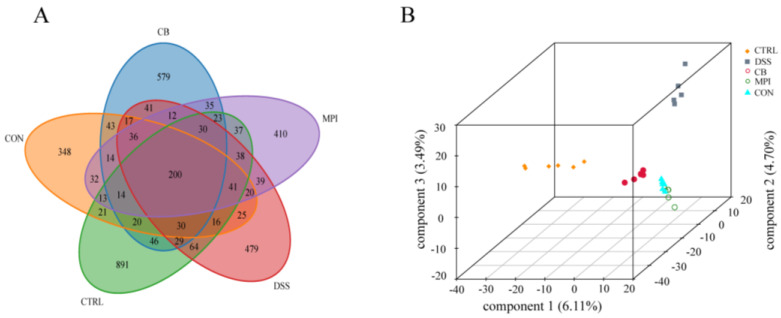
Effects of CB, MPI, and CON on β-diversity of intestinal microbes in mice. (**A**) Venn chart; (**B**) PLS-DA 3D chart.

To investigate whether CB, MPI, and CON could regulate the diversity of intestinal microbes in the DSS-induced colitis mice, colon contents were collected for 16S rRNA gene high-throughput sequencing analysis. The uniqueness and similarity of different samples were represented by a Venn diagram (Figure 5A). The number of OTUs in the CTRL, DSS, CB, MPI, and CON groups was 891, 479, 579, 410, and 348, respectively, and the common number of OTUs of the five groups was 200, indicating that the types and quantities of microorganisms in the five groups were different. Then, the alpha diversity and beta diversity were further analyzed. The DSS group had decreased Chao1 and ACE indices compared with the CTRL group (*p* < 0.05), indicating that DSS would lead to a decrease in the number of species in the colon (Table S3). However, there was no significant difference after CB, MPI, and CON treatment, compared with the DSS group, indicating that CB, MPI, and CON could not significantly change the abundance of intestinal microorganisms. Analysis of the Shannon and Simpson indices showed that the treatment of CB, MPI, and CON did not change species diversity. These findings showed that CB, MPI, and CON have certain limits on the regulation of species richness and diversity. The differences in gut microbial diversity among the five groups were compared by beta-diversity analysis. The partial least-squares discriminant analysis (PLS-DA) showed that there were differences in the intestinal microbes of the five groups of mice (Figure 5B).

**Figure 6:**
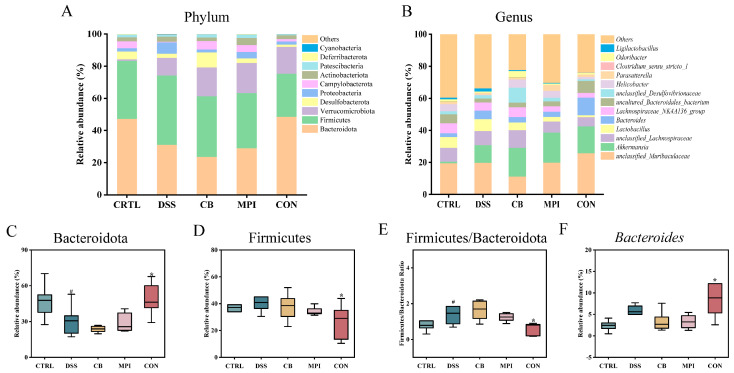
Effects of CB, MPI, and CON on gut microbiota abundance in five groups of mice. (**A**) phylum level; (**B**) genus level; (**C**) Bacteroidota; (**D**) Firmicutes; (**E**) Firmicutes/Bacteroidota ratio; (**F**) Bacteroides. Compared with CTRL, # *p* < 0.05; compared with DSS, * *p* < 0.05.

Concurrently, the effects of CB, MPI, and CON on microbial abundance were studied at different taxonomic levels. The main bacteria at the phylum level were Bacteroidota, Firmicutes, Verrucomicrobiota, and Desulfobacterota, accounting for over 85% of all bacteria (Figure 6A). The proportion of Bacteroidota was 47.20%, 31.06%, 23.64%, 28.97%, and 48.50%, and this indicated that treatment of CON significantly increased the relative abundance of Bacteroidota and was close to the level of the CTRL group. The proportion of Firmicutes was 36.14%, 43.11%, 37.69%, 34.30%, and 26.83%. The main bacteria at the genus level were *unclassified_Muribaculaceae*, *unclassified_Lachnospiraceae*, *Lactobacillus*, and *Bacteroides* (Figure 6B). Compared with the CTRL group, treatment with DSS reduced the relative abundance of Bacteroidota and increased the ratio of Firmicutes/Bacteroidota (*p* < 0.05). Compared with the DSS group, CON treatment increased the relative abundance of Bacteroidota and *Bacteroides*, but decreased the relative abundance of Firmicutes and the ratio of F/B (*p* < 0.05) (Figure 6C–F).

**Figure 7:**
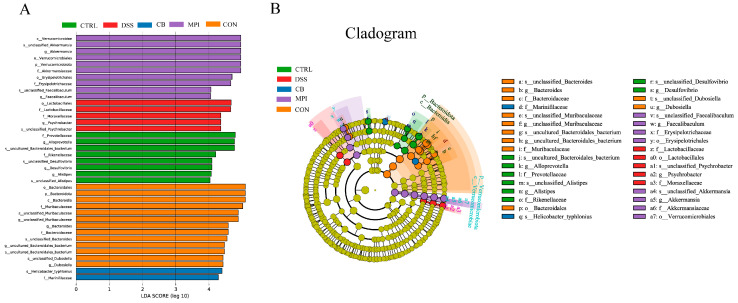
Effects of CB, MPI, and CON on LEfSe analysis of intestinal flora in mice. (**A**) LDA chart; (**B**) LEfse chart.

To understand the differences between groups, linear discriminant analysis (LDA) effect size (LEfSe) was used to identify microorganisms with rich differences between groups. As shown in Figure 7, the dominant bacteria in the CTRL group were Prevotellaceae, *Alloprevotella*, and *uncultured_Bacteroidales_bacterium*; the dominant bacteria in the DSS group were Lactobacillales, Moraxellaceae, and Psychrobacter; the dominant bacteria in the CB group was Marinifilaceae; the dominant bacteria in the MPI group were Verrucomicrobiae, *Akkermansia*, and Erysipelotrichales; and the dominant bacteria in the CON group were Bacteroidota, Muribaculaceae, and Dubosiella. These results suggested that CB, MPI, and CON could change the composition and structure of intestinal flora in mice with DSS-induced colitis.

### 3.5. Serum Metabolite Analysis

**Figure 8:**
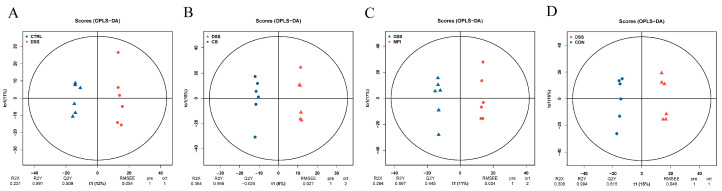
Plots of the multivariate statistical comparisons between groups. OPLS-DA score chart of (**A**) CTRL-DSS, (**B**) DSS-CB, (**C**) DSS-MPI, and (**D**) DSS-CON.

The orthogonal PLS-DA (OPLS-DA) was used to analyze the metabolomic characteristics of serum samples from the mice to understand the differences between the groups (Figure 8). OPLS-DA showed that there was a separation of metabolic profiles between the five groups, indicating a difference in serum metabolites between the five groups.

**Figure 9:**
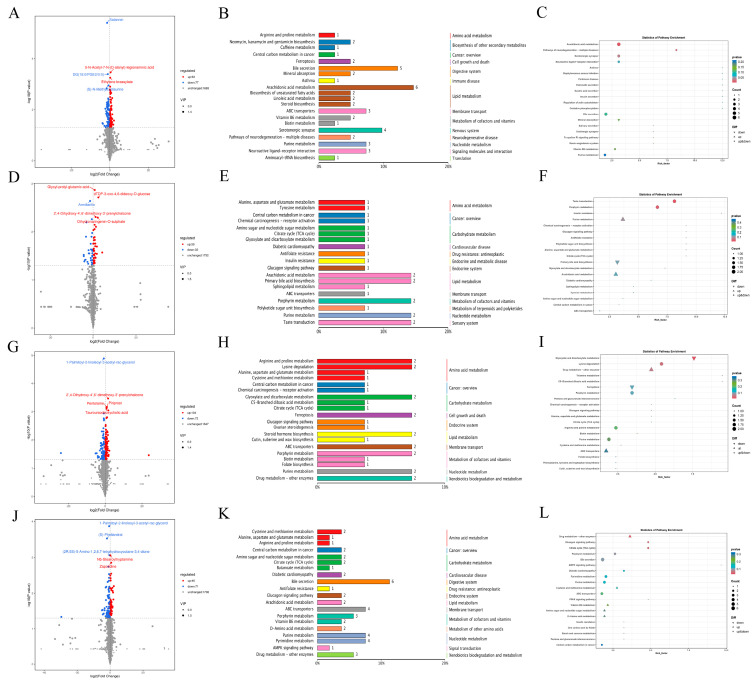
Analysis of differential metabolites in mouse serum. Volcanic chart of differential metabolites of (**A**) CTRL−DSS, (**D**) DSS−CB, (**G**) DSS−MPI, and (**J**) DSS−CON. Differential metabolite pathway classification chart of (**B**) CTRL−DSS, (**E**) DSS−CB, (**H**) DSS−MPI, and (**K**) DSS−CON. KEGG enrichment chart of differential metabolites of (**C**) CTRL−DSS, (**F**) DSS−CB, (**I**) DSS−MPI, and (**L**) DSS−CON.

A total of 1823 metabolites were detected by LC-MS (Figure 9). Comparing the DSS group to the CTRL group, 63 metabolites were up-regulated, 77 metabolites were down-regulated. The Kyoto Encyclopedia of Genes and Genomes (KEGG) database annotation revealed that these differential metabolites were mainly related to bile secretion, arachidonic acid metabolism, and serotonergic synapse. Comparing the CB group to the DSS group, 39 metabolites were up-regulated and 32 were down-regulated; these differential metabolites were mainly related to arachidonic acid metabolism and primary bile acid biosynthesis. Comparing the MPI group to the DSS group, 46 metabolites were up-regulated and 71 metabolites were down-regulated; these differential metabolites were mainly related to the process of steroid hormone biosynthesis and arginine and proline metabolism. Comparing the CON group to the DSS group, 104 metabolites were up-regulated and 72 metabolites were down-regulated; these differential metabolites were mainly related to bile secretion, abc transporters, and pyrimidine metabolism.

Table S4 shows the results of further analysis of the differential metabolites. Compared with the CTRL group, the DSS group reduced content of metabolites such as dihydronaringin-O-sulphate, dodecenoylcarnitine, and (6Z)-Oct-6-enedioylcarnitine and increased content of metabolites such as docosanedioic acid, pitavastatin, 1-octadecanethiol, 3-oxocholest-4-en-26-oate, and 3-keto-4-methylzymosterol (*p* < 0.05). However, compared with the DSS group, the content of metabolites such as *N*-palmitoylsphingosine, guanosine, and dioctyl phthalate increased, and the content of metabolites such as aconitic acid, citric acid, L-urobilinogen, and tilmicosin reduced in the CB, MPI, and CON group (*p* < 0.05). These results indicated that CB, MPI, and CON could change the content of metabolites in mice with DSS-induced colitis.

### 3.6. Correlation Analysis

**Figure 10:**
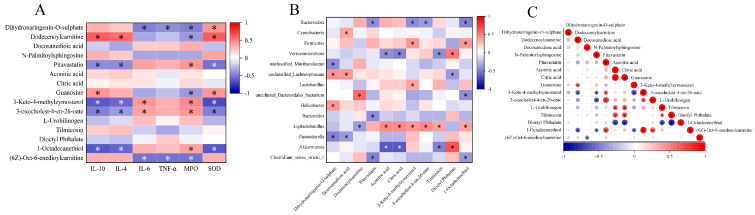
Correlation analyses. (**A**) Correlations between intestinal inflammation−related index and differential metabolites; (**B**) correlations between intestinal flora and differential metabolites; (**C**) correlations between differential metabolites in serum. Red is a positive correlation, and blue is a negative correlation, * *p* < 0.05.

Subsequently, correlation analysis was conducted to explore the connection between intestinal inflammation-related index, intestinal flora, and differential metabolites (Figure 10). The results showed that the concentrations of IL-4, IL-10, and SOD were positively correlated with the content of dodecenoylcarnitine and negatively correlated with the content of 3-keto-4-methylzymosterol and 1-octadecanethiol (*p* < 0.05). The concentrations of IL-6, TNF-α, and MPO were negatively correlated with the content of (6Z)-Oct-6-enedioylcarnitine (*p* < 0.05). The abundance of *uncultured_Bacteroidales_bacterium* was positively correlated with the content of dodecenoylcarnitine (*p* < 0.05). In addition, the content of aconitic acid and citric acid were positively correlated with the content of L-urobilinogen and tilmicosin and negatively correlated with dioctyl phthalate (*p* < 0.05).

## 4. Discussion

UC is an intestinal disease affected by many factors, and its specific pathogenesis is still unclear. Although there are many drugs used to treat UC, some of them are expensive and have side effects. Therefore, the search for safer and more effective treatment has become one of the research hotspots. CB and various active ingredients of PI have been shown to improve colitis [22,23,24]. However, few studies have directly investigated the anti-inflammatory activity of MPI. At present, dozens of different animal models of colitis have been established. Compared with other animal models of colitis, the DSS-induced colitis model has become a useful model for exploring the mechanism related to intestinal inflammation due to its advantages of simple operation, short cycle, and repeatability. However, the pathogenesis of human UC is complex and involves many aspects; the DSS-induced UC model may not fully simulate the characteristics of human UC. DSS induces intestinal inflammation by destroying the colon epithelium, leading to increased permeability of the epithelial cells and allowing the enteral bacteria and bacterial antigens to enter the mucosa [25,26,27]. Using the DSS model, this study compared the repair effects of the CB, MPI, and CB combined with MPI. The results showed that CB, MPI, and CON could effectively reverse weight loss and colon length shortening, reduce colon tissue damage, regulate the balance of inflammatory factors in the colon, improve the composition of intestinal flora and the level of metabolic, and, thus, play a role in relieving colitis. Among them, the comprehensive treatment effect of the CON group was better than the CB and MPI groups.

In this experiment, drinking water was added to 3% DSS, which the mice drank for 7 days. The mice exhibited similar symptoms to human colitis patients [28], such as sparse hair, weight loss, and shortened colons, etc., indicating that this study successfully established a DSS-induced colitis model in mice. After treatment by CB, MPI, and CON, symptoms such as weight loss and shortened colon length were treated, and the severity of colon tissue injury was improved, especially the degree of recovery of crypts. These results collectively support that CB, MPI, and CON intervention could effectively alleviate associated inflammation. Among them, CON had better effects than CB and MPI.

Abnormal activation of NF-κB is widely believed to be the key cause of IBD induction [29]. Dysregulation of NF-κB leads to an increase in gene expression of various genes associated with IBD, including proinflammatory cytokines, enzymes, and genes that regulate intestinal barrier permeability [30]. CD4 Th lymphocytes infiltrate mucous membranes in the body of IBD patients. Naive CD4 Th cells differentiate into different subpopulations when stimulated by particular cytokines: Th1, Th2, Th17, and a subpopulation of regulatory T cells (Tregs) [29]. Th1 cells produce two key proinflammatory cytokines, IL-6 and TNF-α, which are involved in various immune responses [31]. TNF-α is involved in the inflammatory response of lamina propria in IBD patients and increases the permeability of intestinal barrier cells [32]. IL-6 can activate STAT3, and overactivation of STAT3 leads to gastrointestinal injury. Consistent with the results of previous studies [33], DSS treatment in this study increased the concentrations of IL-6 and TNF-α in colon tissue. Th2 lymphocytes secrete two important anti-inflammatory cytokines, IL-4 and IL-10, which restore intestinal permeability by reducing the concentration of proinflammatory cytokines [34]. IL-4 can counteract the appearance of Th1 inflammatory factors (IFN-γ and IL-2) [35], and IL-10 inhibits the production of other inflammatory cytokines by blocking the activation of NF-κB [36]. Results showed that CB, MPI, and CON significantly reduced the concentrations of IL-6 and TNF-a in the colon of DSS-induced mice, and significantly increased the concentrations of IL-4 and IL-10, and the effect of the CON group was better than of CB and MPI. It is suggested that CB, MPI, and CON have immunomodulatory ability and could regulate the balance of proinflammatory and anti-inflammatory cytokines in the colon. Compared with CB and MPI, CON has stronger immunomodulatory ability. For patients with IBD, preventing the abnormal activation of NF-κB is a valid way to treat colitis.

Oxidative stress is a key factor in the occurrence and persistence of IBD-related diseases [37]. In a state of oxidative stress, free radical production greatly increases and exceeds the ability of the body to remove free radicals [38]. Excessive free radicals increase peroxides in tissues, resulting in the destruction of the structure of cell membranes and aggravated tissue damage [39]. MPO is produced primarily by neutrophils and is considered one of the markers of oxidative stress. During inflammation, the inflammatory cascade causes neutrophils to migrate, thereby releasing MPO [40,41]. Many free radicals can cause oxidative stress damage, and SOD can make a difference by declining oxidative stress [42]. Therefore, the concentrations of MPO and SOD were measured in this article to evaluate the degree of oxidative stress in UC mice. The results showed that CB, MPI, and CON significantly reversed the decrease in SOD concentration and the increase in MPO concentration induced by DSS. Compared with CB and MPI, CON had a stronger protective effect against oxidative stress damage during colitis in mice.

The intestinal microecosystem is a complex microbial community [43]. In the gut, different flora form a microbial barrier that maintains intestinal immune homeostasis and prevents pathogenic bacteria from invading [44]. In the human gut, the microbiota is predominantly composed of four main phyla: Firmicutes, Bacteroides, Actinomyces, and Proteus. Firmicutes and Bacteroides are mainly concentrated in the colon, and Actinomyces and Proteus are concentrated in the ileum [44]. However, when the type or quantity of intestinal flora changes, homeostasis of the intestinal environment will be affected, which can result in the occurrence of disease. Therefore, studying the changes in the intestinal flora of UC patients and determining the therapeutic methods to restore the balance of intestinal flora have certain significance for the treatment of UC. In this study, 16S rRNA gene sequencing was used to analyze the microbial community structure in the gut. The dominant bacteria in the five groups of mice were Bacteroidota, Firmicutes, Verrucomicrobiota, and Desulfobacterota. At the phylum level, DSS caused changes in gut microbes, with the abundance of Bacteroidota decreasing. Studies have reported that Bacteroidota was negatively correlated with UC activity indicators, and the aggravation of UC will lead to the disappearance of these species [45]. After CON treatment, the abundance of Bacteroidota increased and Firmicutes decreased; this finding was similar to that reported by Xu et al. [46]. F/B value effectively reflect the imbalance of the intestinal flora [47]. The F/B value of the DSS group was higher than that of the CTRL group, which may be a cause of colitis induction, while the F/B value decreased after CON intervention. The result was similar to previous research studies [47], but there are some studies with opposite results [48]. A previous study showed that *Bacteroides*, an important genus of Bacteroidota, improved lipid metabolism abnormalities caused by dietary fiber deficiency and played a key role in inhibiting colon inflammation [49]. In addition, *Bacteroides* is one of the largest contributors to propionate production in the human gut [50]. These results indicated that CON administration could regulate intestinal microbial composition and improve intestinal inflammation.

Changes in microbes in the gut can lead to metabolite changes; therefore, metabolite changes in the serum of the DSS-induced colitis mice were analyzed using LC-MS. CON treatment reduced the content of metabolites associated with oxidative stress, including L-urobilinogen, citric acid, and tilmicosin. CON treatment increased the content of dodecenoylcarnitine and guanosine. Correlation analysis showed that dodecenoylcarnitine content was positively correlated with IL-4, IL-10, and SOD concentrations. Carnitine can transfer long-chain fatty acids to the mitochondria for β-oxidation, thus playing a role in energy metabolism [51]. Guanosine is a purine nucleoside that has a variety of neuroprotective and cell signal transduction functions. This study suggested that CON intervention could regulate the content of metabolites and reduce intestinal inflammation.

In summary, whether in improving mice weight, colon length, or colon damage, the comprehensive therapeutic effect of CON is superior to CB and MPI. On the one hand, CON may improve the imbalance of inflammatory cytokines and restore intestinal permeability by regulating Th1 and Th2 cells; on the other hand, CON reduced oxidative stress damage by regulating the concentrations of MPO and SOD; on the other hand, CON improved intestinal inflammation by regulating the composition of intestinal flora, increasing the relative abundance of SCFAs producing bacteria; finally, CON also reduced intestinal inflammation by regulating contents of metabolites. After Europe banned the use of antibiotic growth promoters in animal feed in 2006, China banned the addition of antibiotics in pig feed in 2020 [52,53]. Therefore, in view of the advantages of CON in regulating intestinal flora and regulating immunity, CON can be considered as a feed additive. CON can stimulate the proliferation of beneficial bacteria in the gut, improve the utilization rate of CB and MPI, reduce resource waste, and environmental pollution. However, there are also shortcomings in the experiment, and the specific mechanism by which CON improves colitis is still unclear. Therefore, it is valuable to explore the specific mechanism by which CON regulates colitis in the future.

## 5. Conclusions

In conclusion, CON not only reduces inflammation by reducing IL-6 and TNF-a concentrations, but also improves tissue damage by repairing colon structure. In addition, CON could maintain gut health by regulating gut microbes and metabolites. The combination effect of CB and MPI was stronger than using any one component alone. These results suggested that CON has the potential to treat UC and related intestinal disorders. However, further research is needed to investigate the specific mechanism by which CON alleviates colitis.

## Data Availability

The raw sequence data in this study are uploaded to the NCBI database; the accession is PRJNA1001169 (https://www.ncbi.nlm.nih.gov/sra/PRJNA1001169, (accessed on 29 December 2023)).

## Supplementary Materials

The following supporting information can be downloaded at: https://www.mdpi.com/article/10.3390/nu16010153/s1, Figure S1: Effects of CB, MPI, and CON on colon length in colitis mice; Table S1: Histological scoring criteria for colon sections; Table S2: Detailed ELISA kit information; Table S3: Effects of CB, MPI, and CON on the alpha diversity of colon microorganisms in mice; Table S4: Effects of CB, MPI, and CON on serum differential metabolites in mice with colitis.
