# Supplementary material for: Effect of the Combination of Clostridium butyricum and Mycelium of Phellinus igniarius on Intestinal Microbiota and Serum Metabolites in DSS-Induced Colitis
Source: Nutrients. 2024 Jan 2;16(1):153. doi: 10.3390/nu16010153 (PMC10780906; doi:10.3390/nu16010153)
Supplement: Supplementary file 1 [file nutrients-16-00153-s001.zip › nutrients-2739386-supplementary.pdf]

nutrients

MDPI

Article

Effect of the Combination of *Clostridium butyricum* and Mycelium of *Phellinus igniarius* on Intestinal Microbiota and Serum Metabolites in DSS-induced Colitis

Rou Zhao[1], Qiaoyi Zhang[2], Ting Huang[2], Yun Tian[2], Guiping Guan[2], Yuanshan Lin[1,*]

[1] College of Bioscience and Biotechnology, Hunan Agricultural University, Changsha, China
[2] Agricultural Bioengineering Institute, Changsha, China
[*] Correspondence: Wallslin@hunau.edu.cn; Tel.: +86 -139-7487 -6598

1. *Effects of CB, MPI, and CON on colon length in colitis mice*

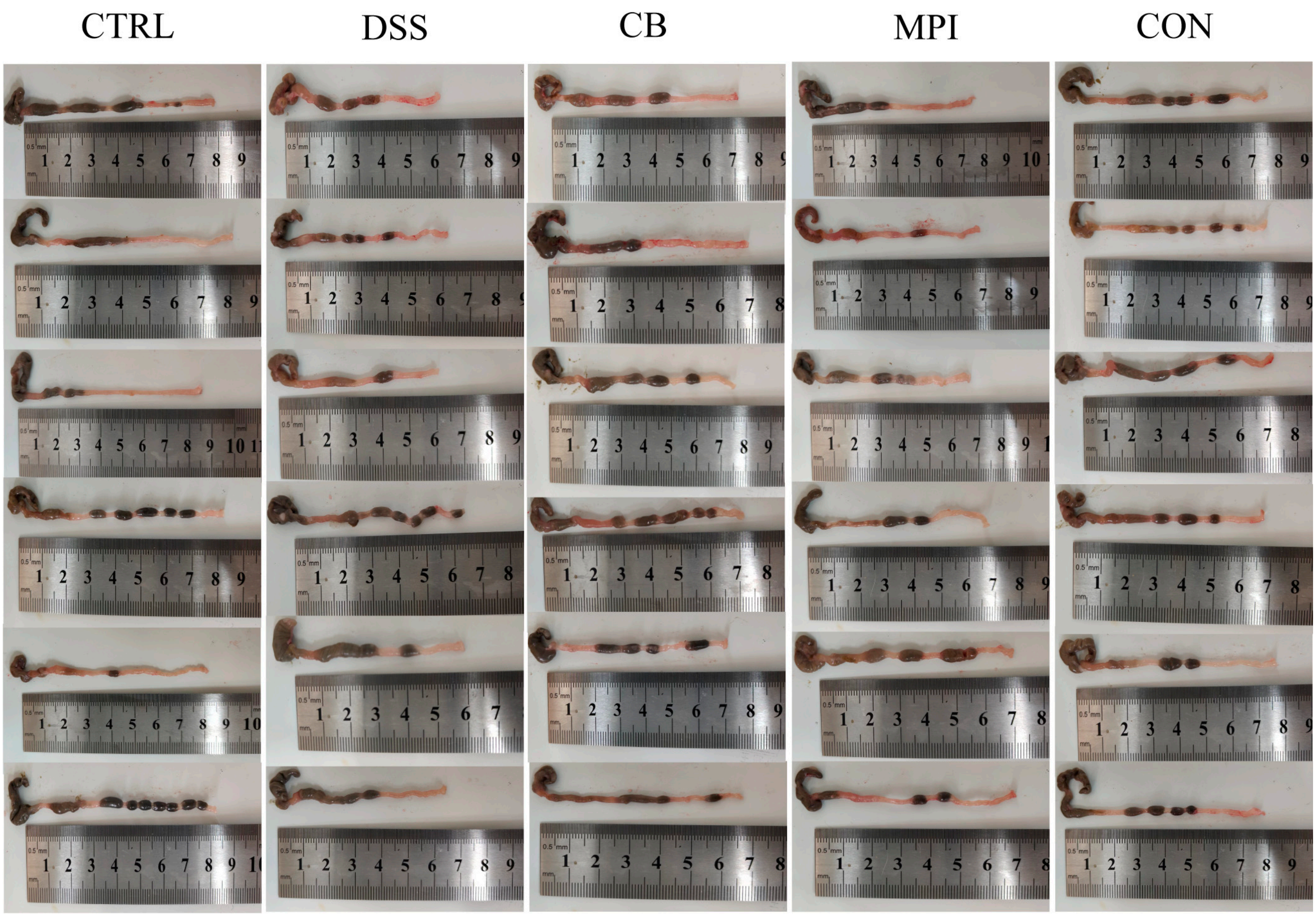

Supplementary Figure S1. Comparison of colon length in each group of mice.

2. *Histological scoring criteria for colon sections*

Supplementary Table S1. Histological scoring criteria for colon sections

Feature graded	Score	Description
	0	None
	1	Minimal
Inflammation	2	Mild
	3	Moderate
	4	Severe
	0	None
	1	Mucosal
Extent of injury	2	Mucosal and submucosal
	3	Transmural
	4	Chorion layer
	0	None
	1	Basal 1/3 damaged
Crypt damage	2	Basal 2/3 damaged
	3	Only the surficial epithelium is intact
	4	The entire crypt and epithelium lost
	0	0
	1	1%-25%
Extent of section affected	2	26%-50%
	3	51%-75%
	4	76%-100%

3. *Detailed ELISA kit information*

Supplementary Table S2. Detailed ELISA kit information

Kit name	Detection range	Manufacturing or marketing company	Country	Kit lot number
IL-4 detection kit	10-240 pg/mL	Jiangsu MeiMian Industrial Co., Ltd	China	20230507M
IL-6 detection kit	3-120 pg/mL	Jiangsu MeiMian Industrial Co., Ltd	China	20230507M
IL-10 detection kit	30-1000 pg/mL	Jiangsu MeiMian Industrial Co., Ltd	China	20230507M
TNF-α detection kit	25-800 ng/L	Jiangsu MeiMian Industrial Co., Ltd	China	20230507M
MPO detection kit	1-40 ng/L	Jiangsu MeiMian Industrial Co., Ltd	China	20230507M
SOD detection kit	1-50 pg/mL	Jiangsu MeiMian Industrial Co., Ltd	China	20230507M

nutrients MDPI

4. *Effects of CB, MPI, and CON on the alpha diversity of colon microorganisms in mice*

Supplementary Table S3. Effects of CB, MPI, and CON on the alpha diversity of colon microorganisms in mice

Index	CTRL	DSS	CB	MPI	CON
ACE	430.47 ± 56.38	327.07 ± 42.62*	318.18 ± 68.02	305.76 ± 50.21	257.47 ± 36.13
Chao 1	429.37 ± 56.35	326.13± 42.48*	317.01 ± 67.79	304.25 ± 50.09	256.60 ± 35.98
Simpson	0.97 ± 0.01	0.95 ± 0.03	0.94 ± 0.17	0.96 ± 0.02	0.96 ± 0.02
Shannon	6.65 ± 0.23	6.17 ± 0.67	5.77 ± 0.47	6.15 ± 0.51	5.88 ± 0.54
PD_whole_tree	32.78 ± 3.63	27.22 ± 2.40	27.80 ±5.04	26.66 ± 3.95	23.27 ± 1.74

Compared to CTRL group, *$p < 0.05$.

5. *Effects of CB, MPI, and CON on serum differential metabolites in mice with colitis*

Supplementary Table S4. Differential metabolites in serum

Metabolite	m/z	CTRL vs DSS		Regulated	DSS vs CB		Regulated	DSS vs MPI		Regulated	DSS vs CON		Regulated
		log2FC	VIP		log2FC	VIP		log2FC	VIP		log2FC	VIP	
Dihydronaringenin-O-sulphate	353.03	−1.73	1.98	↓	2.17	2.81	↑	0.46	0.70	/	1.23	1.76	↑
Dodecenoylcarnitine	376.23	−0.31	1.87	↓	0.32	2.37	↑	0.21	1.39	/	0.45	1.91	↑
Docosanedioic acid	369.30	0.63	2.13	↑	0.09	0.44	/	-0.97	2.38	↓	-0.50	1.89	↓
N-Palmitoylsphingosine	576.50	-0.49	1.06	/	0.41	2.22	↑	0.46	2.06	↑	0.46	1.60	↑
Pitavastatin	466.17	0.41	2.03	↑	-0.17	0.11	/	-0.29	1.57	/	-0.55	1.67	↓
Aconitic acid	173.01	-0.18	1.25	/	-0.36	2.14	↓	-0.73	2.27	↓	-0.36	1.68	↓
Citric acid	191.02	-0.11	1.16	/	-0.45	2.49	↓	-0.67	2.36	↓	-0.48	1.94	↓
Guanosine	301.13	-0.53	1.27	/	-0.71	2.54	↑	0.59	1.97	↑	0.59	1.69	↑
(6Z)-Oct-6-enedioylcarnitine	629.33	-0.46	1.72	↓	0.10	0.62	/	0.26	1.33	/	0.39	1.63	↑
1-Octadecanethiol	331.27	0.76	1.98	↑	-0.34	1.41	/	-0.40	1.49	/	-0.94	1.67	↓
L-Urobilinogen	561.36	0.12	0.36	/	-1.09	2.25	↓	-1.15	1.86	↓	-1.56	1.78	↓
Tilmicosin	886.61	-1.49	1.71	/	-3.72	2.32	↓	-3.85	1.95	↓	-3.99	1.73	↓
Dioctyl Phthalate	413.27	0.25	1.70	/	0.21	2.26	↑	0.29	2.43	↑	0.30	2.08	↑
3-oxocholest-4-en-26-oate	414.31	0.81	1.63	↑	-0.33	1.15	/	-0.44	1.27	/	-0.98	1.59	↓
3-Keto-4-methylzymosterol	361.32	0.88	1.68	↑	-0.69	1.89	/	-0.60	1.62	/	-1.14	1.87	↓

CTRL vs DSS, the control group compared with the DSS group; DSS vs CB, the DSS group compared with the CB group; DSS vs MPI, the DSS group compared with the MPI group; DSS vs CON, the DSS group compared with the CON group. Fold change (FC), indicates the ratio of average metabolite levels between the groups. VIP, variable importance in projection. ↑ / ↓ , increase/decrease, /, no changes. The screening criteria for differential metabolites were set at VIP > 1.50 and $p < 0.05$.
